# Anthropometric Measures as Predictive Indicators of Metabolic Risk in a Population of “Holy Week *Costaleros*”

**DOI:** 10.3390/ijerph16020207

**Published:** 2019-01-13

**Authors:** José Miguel Robles-Romero, Eduardo J. Fernández-Ozcorta, Juan Gavala-González, Macarena Romero-Martín, Juan Gómez-Salgado, Carlos Ruiz-Frutos

**Affiliations:** 1 Nursing Department, University of Huelva, 21007 Huelva, Spain; josemiguelroblesromero@gmail.com; 2 EMOTION Research Group (HUM-643), University of Huelva, 21007 Huelva, Spain; ozcorta@escueladeporte.com; 3 Physical Education and Sports Department, University of Seville, 41018 Seville, Spain; jgavala@us.es; 4 Centro Universitario de Enfermería Cruz Roja, Universidad de Sevilla, 41008 Seville, Spain; mromero@cruzroja.es; 5 Nursing Department, University of Huelva, 21007 Huelva, Spain; 6 Safety and Health Posgrade Program, Universidad Espíritu Santo, Guayaquil 091650, Ecuador; 7 Preventive Medicine and Public Health, University of Huelva, 21007 Huelva, Spain; frutos@uhu.es

**Keywords:** body fat, cardiovascular risk, *costalero*, health prevention, physical endurance

## Abstract

Preventive measures are a priority in those groups that perform intense physical efforts without physical preparation and that can also be overweight or obese. One of the groups that reflect these characteristics is the *costaleros* of the Holy Week of Andalusia, Spain. This paper aims to describe the effect of obesity on blood pressure. A descriptive cross-sectional study was conducted on 101 *costaleros*. The anthropometric measures were determined through segmental impedance. Cardiac recovery and anaerobic power were measured through the Ruffier–Dickson test and the Abalakov test, respectively. Blood pressure was measured when the individuals were at rest. The Kruskal–Wallis test was applied for of continuous parameters and the X^2^ test for dichotomous measures. Binary logistic regression models were used for the subsequent analysis with R-square and Receiver Operating Characteristic (ROC) curves. The average population was 28 years of age, 173.7 cm tall, and 82.59 Kg weigh. The excess of body fat was 11.27 Kg and Body Mass Index was 27.33 Kg/m^2^. 72.3% showed abnormal blood pressure and 68.2% were overweight. 32.7% had a waist-hip ratio higher than 0.94. The probability of presenting abnormal blood pressure was higher among the subjects whose fat content was higher and muscle content was lower.

## 1. Introduction

Arterial hypertension or High Blood Pressure (HBP) is a silent and invisible disease significantly that besets on developed countries, enhancing the occurrence of cardiovascular and cerebrovascular diseases [1]. The increase in blood pressure is mainly associated with structural changes in the arteries, such as stiffness, directly relating to obesity. Obese people have greater fat tissue, which increases, by a mechanical effect, vascular resistance and, at the same time, the burden of cardiovascular work for pumping oxygenated blood to the tissues. Likewise, an increased activation of the sympathetic nervous system happens, as well as changes in the renal structure and function, which is responsible for, among other functions, regulating blood pressure levels [2].

Obesity is caused by the energy imbalance involving both dietary intake and patterns of physical activity [3], known as ‘the big two’ [4] due to their crucial influence on the modification of weight conditions. Caloric intake excess, joined to the deficit of energy expenditure in daily exercise, causes the accumulation of body fat and overweight conditions. To assess these consequences, there are different methods and instruments that have recently related various anthropometric measures, such as the waist-hip ratio (WHR) and the body mass index (BMI) [5] with hypertension, and these may be supplemented by the percentage of body fat [6]. The BMI (weight in kilograms divided by the squared height in centimetres) is the most widely used instrument in epidemiological studies. A normal BMI ranges from 18.5 to 24.9 kg/m^2^, when considering overweight from 25 to 29.9 kg/m^2^ and defining obesity as a BMI ≥ 30 kg/m^2^ [7]. A high BMI is an important risk factor for noncommunicable diseases, such as cardiovascular diseases, diabetes, musculoskeletal disorders, and some types of cancers [8]. Although the BMI can be strongly correlated with some complications, several consequences of obesity may be more strongly associated with the amount of abdominal (or visceral) fat [9], with this tissue being one of the most robust markers of HBP [10]. Another widely used anthropomorphic measurement is the WHR, which has a higher ability of significantly discriminating when compared to the BMI and the hip circumference [11].

Control of body weight, as well as the usual practice of physical activity, are essential pillars in what is known as the healthy lifestyle [8]. Physical activity and, in particular, cardio-respiratory fitness (CRF) or functional capacity can protect against the cardiovascular risk that is associated with obesity [12]. More precisely, obesity and CRF are cardiovascular predictors, and they also predict all causes of death [13,14,15], regardless of other cardiovascular risk factors [16]. Cardio-respiratory fitness is defined as “the ability to perform daily life physical tasks by means of aerobic metabolic processes”. It provides important diagnostic and prognostic information [17] and it even has raised speculation as to whether their score should be included as a risk factor for cardiovascular diseases [18,19].

Cardio-respiratory fitness is explained through the maximum oxygen absorption (VO_2_ max) [20] and it has now been linked to health markers, such as cardiovascular disease and mortality. Individuals with low CRF have 70% more risk of all the causes of mortality and 56% risk of mortality from a cardiovascular disease [21]. Therefore, it is considered useful to know the VO_2_ max when studying parameters that are associated with cardiovascular diseases. The direct evaluation of VO_2_ max presents a number of limitations, such as an increased risk of adverse cardiovascular events, expensive equipment for the measurement of gases, the lack of motivation that the test has proven to show, and the reduction of tolerance to physical exercise [20]. In addition, the maximum exercise criteria are not often met in non-athlete populations [22], existing a large number of submaximal protocols to estimate the variable that can be performed both in laboratories and in health/fitness centres [23]. Among the different submaximal tests, we find the Ruffier-Dickson index (RDI) to classify the cardio-respiratory aptitude, which takes into account both the parameters at rest and recovery and that proves to be an appropriate protocol to provide an easy and quick estimate of the VO_2_ max [24].

Preventive measures are a priority for the general population from the health point of view, but even more so if the collective studied performs intense physical efforts without physical preparation and even more when they can also be overweight and have a low CRF. One of the groups that meets these characteristics is the *costaleros* of the Holy Week of Andalusia, Spain, for making important physical efforts, of special characteristics, for a short period of time, and without prior preparation. Their work is characterised by rising, through anaerobic effort of explosive force and to the voice of a hammer sound, and carrying on their shoulders a metal or wooden structure of an approximate weight of 50 kg for a period longer than 5 h. The structure is raised explosively, being supported on the seventh cervical vertebra, as under the structure there are a few rounded beams that are designed to support the *costalero*’s neck. Subsequently, they have to walk and move the structure during periods of about 3 min, with breaks of 3–4 min in most cases.

We believe that it is useful to determine the amount of body fat in this group and to correlate it with the effects that this causes in the coronary response to physical efforts through the estimation of CRF. The objective of the present study is to know the effect of obesity (measured by the BMI, WHR, and percentage of fat mass) on the blood pressure at rest, taking into account the consumption of tobacco, the level of physical fitness, the RDI, jump height, and percentage of muscle mass are useful.

## 2. Materials and Methods

### 2.1. Participants

A descriptive cross-sectional study was conducted on 101 *costaleros* of the Holy Week of Huelva, Andalusia, Spain. *Costaleros* are the people who lift and carry on their shoulders a religious image in the Holy Week celebrations of Andalusia, Spain. The sample consisted only of men, with all belonging to a single team of *costaleros*. The *costaleros* population of the Holy Week does not have an official census on which calculations can be based, but by documentary sources it is estimated that it must not amount to more than 700, so our sample has important significance in terms of figures. The selection of the same was done while considering the group that greater weight supports and that longer distance covers during the performance of such activity.

### 2.2. Procedure

Information was collected on the bodily characteristics, demographic data and habits of life of the studied subjects on the previous week to Holy Week celebrations. In the same way, physical tests were carried out on each individual, highlighting the cardiac adaptation to the effort test (Ruffier–Dickson) [24].

Our study independent variable, blood pressure at rest, was measured the day that the work was carried out, following the recommendations by Mancia et al. [25]. Values were subsequently recoded according to the new guide on hypertension [26]. The blood pressure cuff Riester Minimus II (Rudolf Riester, Germany) and a stethoscope were used for the variable measurement. The analysed dependent variables that could be related to the increase in blood pressure figures were calculated through the bioelectrical impedance analysis, using the multifrequency analyser Inbody 230 (Biospace Corp., Seoul, Korea). This is a segmental impedance device that uses an eight-point tetrapolar tactile electrode method. Ten measurements of impedance were performed using two different frequencies (20 and 100 kHz) in each segment (right arm, left arm, trunk, right leg, and left leg). The subjects were measured in underwear and without socks. The participants were kept on the analyser while the device measured their body weight and the identification number, age, sex, and height of the subjects was entered into the machine. The impedance was measured with the subject still and holding hand grips. The obtained variables were:

- Body mass index (BMI): with the participant standing upright, with bare feet, and with his head at the height level, it was calculated to the nearest 1mm using the portable stadiometer Seca 217 (Medical Measuring Systems and Scales, Germany). The BMI is calculated by dividing the weight (in kilograms) by the height (in square metres) and categorising the sample according to the World Health Organization classification, being under-weight: <18.5 kg/m^2^; standard weight: 18.5–24.9 kg/m^2^; overweight: 25–29.9 kg/m^2^; and obese ≥ 30 kg/m^2^.
- Body fat percentage and muscle mass percentage: Data output, calculated by the manufacturer’s algorithm, includes the amount of body fat measured in Kg as well as the percentage of tissue at body level. The normality data of this variable and the following ones were obtained according to the recommendations of the Spanish Society for the Study of Obesity (SEEDO) [8].
- For the WHR calculation, manual measurements of these circumferences were performed on all participants bare chested. Normality and non-normality figures were calculated through studies analysed in the previous revision [11].

It should be noted that all of the participants were in previous fasting of 3 h (including fluids), had urinated before the impedance analysis, and had not performed any physical activity before the test.

The physical tests performed offered a general view of the individuals’ adaptation to physical efforts. For this, a physical test was performed for the assessment of the anaerobic resistance (Ruffier–Dickson) and another one for the anaerobic power (Abalakov):

- The Ruffier–Dickson squat test consisted of a 45-s squat exercise (40 pushups/min), followed by a 3-min recovery period. The parameters assessed were: resting heart rate before the squats exercise, heart rate at the end of the exercise, and recovery heart rate after 1 and 3 min. In addition, the RDI was calculated according to the following equation: RDI = (P1 − 70) + 2 (P2 − P0)/10 [27]. According to this equation, P0 is the resting heart rate after 15 s, P1 is the maximum heart rate recorded during the first 15 s of recovery, and heart rate is the average of 15 s after the first minute of recovery (the period from 1 min and 00 s to 1 min and 15 s) [24].
- To know the anaerobic power of the sample, the Abalakov test was performed [28]. This test consists of evaluating the ability to jump by the flexion-extension of the legs in a coordinated manner and synchronised with the action of the arms. This test was carried out, prior to the action of the jump, with the subjects in a bipedal position with a free position of hands and arms, in order for the limbs to be used in a coordinated way.

Behavioural measures include smoking status (never, current, or ex-smoker) and physical activity level, categorised as active (≥2.5 h/week of moderate physical activity or ≥1 h/week of vigorous physical activity), moderately active (<1 h/week of moderate physical activity and <1 h/week of vigorous physical activity), or inactive (not active or inactive). The consumption of fruits and vegetables was evaluated with the question “How often do you eat fresh fruits or vegetables?”. The answers were given on a two-point scale: ≥1 fruit or vegetable/day, or <1 fruit or vegetable/day.

### 2.3. Statistical Analysis

The statistical analysis was carried out with the statistical programme SPSS Ver. 23 (IBM). The Kruskal-Wallis test was applied for the statistical significance of continuous parameters differences between groups of *costaleros,* and the X^2^ test for dichotomous measures (tobacco use). Binary logistic regression models were built to identify the factors that were independently related to the presence of abnormal blood pressure. The variables included in the model were: BMI, WHR, percentage of fat, and percentage of muscle mass. Also, the Nagelkerke R-square was estimated to indicate the dependent variable’s part of the variance explained by the used model. The null hypothesis was rejected in each statistical test with a *p* < 0.05 value.

Non-parametric Receiver Operating Characteristic (ROC) curves were used to classify people with normal/abnormal blood pressure through the goodness-of-fit test by Hosmer and Lemeshow to confirm the proposed model adjustment.

### 2.4. Ethical Considerations

All the participants were informed of the study, prior to voluntary acceptance, and signed a consent. Data was recorded maintaining the anonymity of participants. The study is in line with the latest version of the Helsinki declaration. It has been approved by the Research Ethics Committee of Huelva, Spain (ID: PI 086/15).

## 3. Results

The *costalero* prototype is a young man aged between 18–51 years of age (28.86 ± 8.63), with an average height and weight of 173.7 ± 5.74 cm and 82.59 ± 14.71 Kg, respectively. An excess of body fat of 11.27 kg, a BMI of 27.33 kg/m^2^, and a basal caloric consumption of 1677 kcal/day categorises this group as obese.

72.3% (72) of the studied population showed abnormal blood pressure (Systolic Blood Pressure ≥ 120 or Diastolic Blood Pressure ≥ 80) at the time of data collection and 68.2% (69) showed overweight. 32.7% (33) had a WHR higher than 0.94. The amount of accumulated fat, adjusted to the age of the subjects, was above the recommended for their age in 67.3% (68) of the cases. In Table 1, we see how, while using the results and according to the blood pressure classification, there have been statistically significant differences in the variables weight (H (3) = 17.631; *p* = 0.001), BMI (H (3) = 16.831; *p* = 0.001), percentage of body fat (H (3) = 16.553; *p* = 0.001), percentage of skeletal muscle mass (H (3) = 17.028; *p* = 0.001), and WHR (H (3) = 10.148; *p* = 0.017). On the contrary, there have been no significant differences between the groups according to blood pressure levels with respect to the variables: age, size, smoker, RDI, and height of jump.

Logistic regression showed that the probability of presenting abnormal blood pressure was worse among the subjects with higher fatty percentage (0.15 (0.001–0.347); *p* < 0.009) and lower muscle mass percentage (0.10 (0.00–0.291); *p* < 0.007). However, BMI (*p* = 0.325) and WHR (*p* = 0.794) values were not significant in the model. According to Nagelkerke R-square, the fatty percentage and the muscle mass percentage could explain, in the studied population, 29.6% of people with abnormal blood pressure.

**Figure 1:**
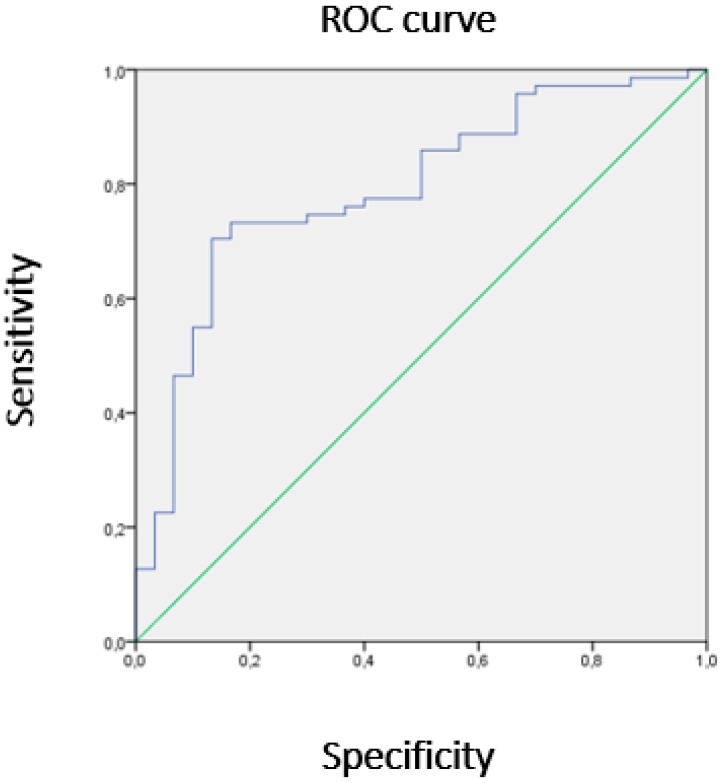
Receiver Operating Characteristic (ROC) curve.The blue line represents the union of the cut points of the diagnostic test, the Y axis corresponds to the sensitivity and the X axis to 1-specificity. Both axes include values between 0 and 1 (0% to 100%). The green line represents the reference diagonal or line of non-discrimination, is plotted from point 0,0 to point 1,1.

Taking into account the fat mass and muscle mass percentages, we see that subjects with abnormal blood pressure (SBP ≥ 120 or DBP ≥ 80) showed an area of 79% (AUC = 0.79) under the ROC curve (Confidence Interval 95%; 0.696–0.886). We can interpret that, from an area of 0.7, the discrimination of the model is considered to be acceptable. On the other hand, the test of the Hosmer and Lemeshow test confirmed that the proposed model fits the observed values (*p* = 0.303) (Figure 1).

## 4. Discussion

This study has allowed us to identify a group with a high percentage (72.3%) of abnormal blood pressure (SBP ≥ 120 or DBP ≥ 80) and a 59.4% of prehypertension. We see how, close to 30 years of age, there is a clear increase in blood pressure and that this increases with age, data that match the Di@bet.es study [29].

Contrary to what is found in the literature, where tobacco consumption and high blood pressure [30] have been related, we have not found this association in our data. However, in other studies it was appreciated that tobacco consumption could decrease blood pressure [31].

High BMI and WHR are associated with an increased risk of cardiovascular disease and mortality [6], revealing that the studied group had a BMI above the level considered normal and healthy [7] in 68.2% of cases, which is higher than the average values of the Andalusian population (60%) [32]. One-third of the group had a higher than normal WHR and individuals with high blood pressure gave a WHR higher than 0.94, having found an association between these two parameters in the literature [5].

When relating the variables body fat and muscle mass with the BMI, we find that there is a direct relationship between them with respect to the index (0.4 and 0.53 kg/m^2^ per 1 raised kg). This shows the little usefulness of this index given that the analysed individuals are people who have a high muscle content (young and/or athletes).

The studied group is subjected to efforts of high physical demand, assessed at a four over five level of hardness, which is in line with previously published data [29,30,31]. Generally, they have no prior physical preparation [27], unlike other groups of workers, such as stevedores or dockers, where such preparation allows for them to acquire an athletic physical build [33].

It is known from the literature that people with high blood pressure subjected to high exercise of more than 9.4 mm Hg per minute of exercise present a high relative risk of myocardium infarction [34]. It has been found how 67.3% (68) of the studied group of *costaleros* presents a high amount of accumulated fat, once adjusted by age. This result is particularly significant, knowing the impact of accumulated fat on blood pressure and, consequently, on the risk of myocardial infarction.

We can consider whether anthropometric values can serve as predicting variables of pathological weight conditions. Knowing the effects of the WHR at the cardiovascular level [35], we found that those participants who had a WHR above 0.94 doubled the risk of suffering abnormal blood pressure figures (72% of those studied), having a 12% level of level 2–3 hypertension. If we consider BMI as a predictor, we see how those who are overweight or obese had a high risk of suffering hypertension, 3.5 times more than those of a normal weight condition, and increasing to 4.5 in those who showed an increase in body fat.

There is a general perception of the studied group’s health as good (4 out of 5), of having a regular physical condition and taking care of their diet both during the year and during the time of carrying out the work, despite referring eating three times a day and despite the high figures of weight they present. In addition, and although they consider the work as hard (4 out of 5), they do not perform any previous preparation for its performance, or put in place appropriate dietary habits that will assure a less harmful execution of the task. They claim to smoke during the day of the task although they do not consider themselves habitual smokers. All of this presents an image of the *costalero* with an excess of weight, an unhealthy lifestyle, and being subjected to a very demanding physical effort without previous preparation.

One of the strengths of our study is the use of such data regarding the direct association between body fat and WHR and increased blood pressure figures by quantifying this increase by index unit. Thus, this process can be generally applied to any population, highlighting both variables as highly relevant cardiovascular risk factors.

As for weaknesses, it is worth mentioning the fact that an exhaustive sampling for this study was not performed. This was not done so, because the complexity of the tests performed and the high number of participants in relation to the existing population led the study to focus on a single collective (one specific brotherhood) and select the one whose work was of greater intensity and physical strain.

## 5. Conclusions

We can conclude that variables, such as body mass index, waist-hip ratio, and body fat level are good predictors of cardiovascular risk, as has already been published in previous studies.

The increase in body fat is inversely correlated with the capacity of cardiac recovery after the effort. If we consider the type of work *costaleros* carry out and its characteristics, we can conclude that they are a group at cardiovascular risk.

Increased body fat levels provoke an increase in blood pressure figures at rest, which confirms the importance of controlling adiposity levels as a risk factor for arterial stiffness in middle-aged adults.

The use of BMI to identify cardiovascular risk groups can be interesting in general, but when dealing with people who have a high content of muscle mass, this can result in the emergence of false positives. These outcomes turn out to not be useful for the classification of people with coronary problems.

The studied group of *costaleros* is subjected to a high physical demand with little previous physical preparation, which increases the cardiovascular risk in the medium and long term.

Although the studied participants perceive their health status as good and believe that they take care of their diet, the facts do not seem to show a healthy lifestyle. All of this categorises them as a risk group from a cardiovascular point of view. The differences between real health and perceived health makes the modification of unhealthy habits more difficult, as it is really complex to achieve this objective if there is no real risk conception on the part of the individual.

## Figures and Tables

**Table 1:** Characteristics of the studied variables regarding blood pressure classification of the sample.

	Blood Pressure Levels					
	Population *n* = 101	Normotension SBP < 120 DBP < 80 (*n* = 29; 28.7%)	Pre-Hypertension SBP = 120 y ≤ 139 DBP = 80 y ≤ 89 (*n* = 60; 59.4%)	High Blood Pressure Stage 1 SBP = 140 y ≤ 159 DBP = 90 y ≤ 99 (*n* = 10; 9.9%)	High Blood Pressure Stage 2 SBP ≥ 160 DBP ≥ 100 (*n* = 2; 2%)	
	M ± SD	M ± SD	M ± SD	M ± SD	M ± SD	*p*
Age (year)	28.89 ± 8.60	26.31 ± 8.21	29.08 ± 8.30	33.20 ± 7.89	39.00 ± 16.97	0.831
Weight (kg)	82.96 ± 14.71	74.48 ± 11.88	84.98 ± 13.47	88.80 ± 14.42	116.30 ± 14.28	0.001
Height (cm)	173.78 ± 5.74	172.25 ± 4.56	174.24 ± 5.93	174.93 ± 6.76	175.55 ± 10.68	0.243
Smoker (yes/no)	1.67 ± 0.47 32.7%	1.72 ± 0.455 27.6%	1.67 ± 0.48 33.3%	1.50 ± 0.53 50%	2.00 ± 0.00 0%	0.452
BMI (kg/m^2^)	27.44 ± 4.41	25.08 ± 3.69	27.99 ± 4.12	28.98 ± 4.06	37.65 ± 0.07	0.001
Fat mass percentage (%)	25.90 ± 7.88	21.61 ± 7.13	26.87 ± 7.48	30.22 ± 7.05	37.40 ± 1.70	0.001
Muscle mass percentage (%)	69.91 ± 7.48	74.04 ± 6.78	68.96 ± 7.08	65.76 ± 6.62	59.16 ± 1.49	0.001
WHR	0.92 ± 0.05	0.90 ± 0.05	0.92 ± 0.05	0.94 ± 0.04	0.99 ± 0.04	0.017
RDI	4.94 ± 2.75	4.67 ± 2.65	5.03 ± 2.99	5.08 ± 1.69	5.20 ± 0.85	0.784
Jump height (cm)	32.66 ± 6.47	33.24 ± 7.01	32.57 ± 6.20	31.80 ± 5.77	31.50 ± 14.85	0.869

SBP = systolic blood pressure; DBP = diastolic blood pressure; WHP = waist-to-hip ration; RDI = Ruffier–Dickson Index; M ± SD = mean and standard deviation.
